# Information Access and Use by Patients With Cancer and Their Friends and Family: Development of a Grounded Theory

**DOI:** 10.2196/20510

**Published:** 2020-10-29

**Authors:** Maclean Thiessen, Shane Sinclair, Patricia A Tang, Shelley Raffin Bouchal

**Affiliations:** 1 Research Institute in Oncology and Hematology CancerCare Manitoba Winnipeg, MB Canada; 2 Department of Internal Medicine Rady Faculty of Health Sciences University of Manitoba Winnipeg, MB Canada; 3 Faculty of Nursing University of Calgary Calgary, AB Canada; 4 Department of Oncology Cumming School of Medicine University of Calgary Calgary, AB Canada

**Keywords:** persons, personal autonomy, patient-centered care, health education, health information–seeking behavior, grounded theory, empowerment, cancer, qualitative research, adaptation, psychological, mobile phone

## Abstract

**Background:**

Information has been identified as a commonly unmet supportive care need for those living with cancer (ie, patients and their friends and family). The information needed to help individuals plan their lives around the consequences of cancer, such as the receipt of health care, is an example of an important informational need. A suitable theory to guide the development of interventions designed to meet this informational need has not been identified by the authors.

**Objective:**

The aim of this study is to generate a grounded theory capable of guiding the development of interventions designed to assist those living with cancer in meeting their informational needs.

**Methods:**

Classic grounded theory was used to analyze data collected through digitally recorded one-on-one audio interviews with 31 patients with cancer and 29 friends and family members. These interviews focused on how the participants had accessed and used information to plan their lives and what barriers they faced in obtaining and using this information.

**Results:**

The theory that emerged consisted of 4 variables: personal projects, cancer as a source of disruption to personal projects, information as the process of accessing and interpreting cancer-related data (CRD) to inform action, and CRD quality as defined by accessibility, credibility, applicability, and framing. CRD quality as a moderator of personal project disruption by cancer is the core concept of this theory.

**Conclusions:**

Informational resources providing accessible, credible, applicable, and positively framed CRD are likely key to meeting the information needs of those affected by cancer. Web-based informational resources delivering high-quality CRD focused on assisting individuals living with cancer in maintaining and planning their personal projects are predicted to improve quality of life. Research is needed to develop and integrate resources informed by this theoretical framework into clinical practice.

## Introduction

### Background

One of the most commonly reported unmet supportive care needs of those facing cancer, including patients [1-4] and their friends and family [5-7], is for information. Common informational needs include those related to prognosis, how to care for someone with cancer, and the benefits and toxicities of treatment [2,8-14]. Unmet information needs are associated with decreased treatment adherence [15,16], increased health care costs [17-19], anxiety [20,21], and depression [20].

In a previous study conducted by the lead author (MT) [22] it was identified that the diagnosis of malignancy resulted in widespread interruption to the lives of those affected by cancer. This study used grounded theory [23,24] to analyze data from 43 semistructured interviews conducted with 18 patients who had been recently diagnosed with cancer and 15 friends and family in Manitoba, Canada. One conclusion from this study was that to support individuals affected by cancer, information is needed that supports them in planning the activities necessary for maintaining participation in the relationships and projects they established before diagnosis—such as those with family, friends, and their work—both in the short and long term. Addressing this finding was a major motivator for the work presented in this study.

Health information–seeking behavior (HISB) is a field of research encompassing how those affected by illness utilize health information. Important areas of study within HISB include how individuals seek, use, and share information [25-27]. The HISB literature can be broadly divided into 3 main categories: (1) coping with health situations, (2) involvement in shared medical decisions, and (3) behavior change and preventative behavior [25,27]. In their concept analysis of HISB, Lambert and Loiselle [27] identified that one goal of the individual engaging in HISB is to better understand what to expect. They identify that HISB serves to “increase predictability” [27] and may assist with “anticipating the sequence of events” [27] that will likely take place. These goals of HISB are consistent with the informational need identified in the study leading up to this work: that information is essential to helping individuals know what to expect in the future so that they can effectively plan how to live their lives [22].

The factors that affect HISB are complex, as illustrated by the examination of the Miller Behavioral Style Scale (MBSS) [26-28] and the model of health information acquisition (HIA) [26,27,29], both of which are utilized in the HISB literature [26,27]. The MBSS is a validated scale [28,30] useful for characterizing individual information-seeking styles in response to both *physical and psychological stress* [28]. The MBSS categorizes individuals as either information seekers (high monitors) or information avoiders (low monitors) [28,30]. High monitors are more likely to seek out information to cope with stressors, whereas the response of low monitors is to avoid information [28,31,32]. The HIA, developed by Freimuth et al [29] from their work with the National Cancer Institute’s telephone-based Cancer Information Service [26], predicts that the decision to seek information involves a cost-benefit analysis. The expected benefit of information is weighed against the effort to seek additional information [26,27,29] in terms of cost considerations such as “financial and time expenditures, frustration, confusion, [and] emotional distress” [29]. Together, the MBSS and the HIA suggest that both intrinsic and external variables impact an individual’s ability to access and use health information to plan around the receipt of health care. This conclusion is supported by the wealth of empirical evidence, including multiple systematic reviews [1,5,7,33-40], consistently correlating specific demographic factors (eg, age, gender, education) [33,41,42] with various types of information-seeking behavior [27,43] and health information needs [5,35].

In the context of cancer, it is not clear how to optimally design interventions to support individuals in obtaining the health information that is most useful for them. In general, using theory to guide intervention design results in better outcomes [44,45]. The application of theory in intervention design facilitates the identification of key constructs to be included in the intervention, potentially resulting in a stronger effect [46]. In addition, the results of testing theory-based interventions provide valuable feedback about the accuracy of the theory [23,46], furthering the understanding of the contextual area under study and facilitating modification of the theory to enhance its accuracy [23].

Multiple theories have been used to guide both the understanding of HISB [26,27] and the development of interventions [37]. Importantly, besides the model of HISB by Longo [47,48] and the HIA by Freimuth et al [29,48], few theoretical frameworks regarding HISB have been developed within the context of cancer. In addition, although existing theories in the HISB literature facilitate understanding and explanations of HISB patterns, the utility of these theories for developing interventions is not clear. For instance, although existing theoretical frameworks employed in the HISB cancer context [29,47-49] describe a cost-benefit relationship in terms of whether an individual will search for additional information, they do not provide guidance in terms of how to structure interventions for those affected by cancer to minimize the cost and maximize the benefit of information seeking. A theoretical framework that addresses this gap in the literature is thought to be valuable for developing interventions that address the informational needs of those living with cancer [1,4,11,12,14,15,35,38,48]. Such a framework would be capable of informing the development of interventions that support individuals in planning their lives around the short- and long-term consequences of cancer, including the receipt of treatment and altered life expectancy [22,50].

### Objectives

The objective of this study is to develop a grounded theory capable of guiding the creation of informational resources designed to assist individuals living with cancer in meeting their informational needs by minimizing the cost and maximizing the benefit of information seeking.

## Methods

### Study Approach

This study used classic grounded theory (CGT), a method for discovering theory through iterative data collection and analysis [51-53]. CGT has been identified as a method for uncovering latent behavioral patterns and generating theory capable of guiding practical action for problem solving [54]. This was one reason that CGT was considered the ideal method for this study as the objective required a theory that operationalized (1) why information related to cancer is important for supporting individuals affected by it and (2) how information about cancer can be optimally provided to improve the lives of those affected by it. Both questions assume that shared patterns of behavior exist among those affected by cancer.

### Study Procedures

The study procedures, including the study design, data collection, analysis, and drafting of the report, were conducted primarily by the lead author, who was completing a medical oncology fellowship during the first year of the study and enrolled in a PhD graduate program as well as in active independent clinical practice as a medical oncologist for the subsequent portion of this study. The second and fourth authors provided methodological support in conducting and presenting the grounded theory analysis. The third author provided general research expertise and contextual expertise regarding clinical oncology practice.

#### Ethical Considerations

Approval for this study was obtained through the Health Research Ethics Board of Alberta (Study ID: HREBA.CC-17-0365) before the initiation of recruitment, data collection, and data analysis.

#### Recruitment

Patients were recruited using posters and invitation letters from a large outpatient cancer facility in Western Canada. Interested patient participants contacted the lead author or primary investigator who provided further details of this study, including its methods, objectives, risks, benefits, and obtained written consent. The patient participants were invited to approach any friends and family to participate in this study as secondary participants. This study was open to all patients aged 18 years or older who had received oncology care. Friends and family participants aged 18 years or older were welcome to participate. Exclusion criteria were limited to not being able to communicate in English and being aged below 18 years. Incentives for participation included being eligible to win one of four Can $25 (US $18.79) gift certificates.

The rationale for inclusion of both friends and family participants as well as patient participants in this study was three-fold. First, it was assumed that, besides instances where patients were receiving medications affecting their cognition or had severe neurological sequelae of their cancer, such as a debilitating brain metastasis, there would be no psychological or sociological phenomena differentiating the processes of information seeking and use for those diagnosed with cancer from their family and friends. Therefore, the concepts and resulting theory that would emerge would likely be valid for both friends and family as well as patients. Second, it is recognized that informal caregivers are often left behind when it comes to supportive care research, including research related to information needs. Although the theory that was expected to emerge would likely be applicable to both groups, without including both patients and friends and family in the study, the validity of the theory for the group not included would likely be questioned. Finally, the contrasting perspectives of friends and family and patients were expected to provide extremely useful data for the purposes of constant comparison, ensuring that theoretical saturation occurred [23,24].

#### Data Collection

After obtaining written consent, all participants completed a demographic questionnaire (Multimedia Appendix 1). A review of the electronic medical charts of patient participants was performed to facilitate the identification of details that were not readily available outside of a thorough interview in the style of detailed medical and treatment history. The data from the chart review and questionnaires were compiled into a database to assist with theoretical sampling, an iterative sampling technique to ensure theoretical coverage and heterogeneity [23,24]. For instance, patients were initially interviewed as they were recruited, resulting in a predominance of patients >50 years of age with breast and colorectal cancer being interviewed. Therefore, the database was used to identify and select participants for interviews who were primarily younger patients with less common malignancies. This was important to ensure that the emerging concepts were adequately informed by data from individuals likely to have had contrasting experiences.

All interviews were semistructured (Multimedia Appendix 2), face to face, and audio recorded. The interviews were carried out in participants’ homes, apart from 3 interviews conducted over the phone. Interviewees were encouraged to stop the interview at any point if they were no longer comfortable proceeding or needed a break; in addition, they were provided with contact information for psychosocial support available through the cancer center. The interviews with participants took place separately, except for 7 interviews where the patients and their friends and family wanted to be interviewed together. Participants were interviewed once; no repeat interviews were conducted. The audio recordings from the interviews were transcribed by a professional transcriptionist as soon as possible after each interview to facilitate ongoing and iterative data analysis. The average interview length was 53 min overall, 1 hour and 4 min for interviews involving patients, and 36 min for interviews with only friends and family participants. Participants who participated in interviews were offered a 24-hour parking pass to the cancer center.

#### Data Analysis

Data analysis using constant comparison was carried out in keeping with the CGT [51]. Data analysis and data collection occurred in an iterative manner, beginning once the first interview was transcribed. Coding, memoing, and theory generation were guided by comparing coded incidents and intentionally selecting participants and interview questions likely to result in data being collected that would contrast with previously collected data, providing new insight to guide the emerging theory [51]. Data collection ceased when data saturation occurred, whereby no new data emerged from the ensuing interviews. The initial stage of coding the collected data (ie, open coding) resulted in a coding schema emerging and the identification of a core category. This facilitated the next stage of the CGT analysis (ie, selective coding), where the theory began to emerge around the coding categories (ie, variables) associated with the consequences of access to the information that participants identified as being helpful or unhelpful. The final stage of the CGT analysis (ie, theoretical coding) involved coding to finalize theoretical links between the categories connecting the challenges faced by the participants, their experience with cancer, and information.

## Results

### Participant Characteristics

A total of 37 patients and 36 friends and family consented to be contacted for interviews, with 31 patients and 29 friends and family (Table 1) completing interviews.

**Table 1:** Interviewed participants’ demographics (n=60).

Interviewed participants				Values
**Interviewed primary participants (n=31)**				
	Age (years), mean (range)			60 (29-81)
	**Gender, n (%)**			
		Male	14 (45)	
		Female	17 (55)	
	**Initially curative at the time of diagnosis, n (%)**			
		Yes	26 (84)	
		No	5 (16)	
	**Currently curative (at the time of interview), n (%)**			
		Yes	19 (61)	
		No	12 (39)	
	**Malignancy type, n (%)**			
		Colorectal	8 (26)	
		Noncolorectal gastrointestinal malignancy	5 (16)	
		Breast	9 (29)	
		Melanoma	5 (16)	
		Hematologic malignancies	3 (10)	
		Osteosarcoma	1 (3)	
**Interviewed secondary participants (n=29)**				
	Age (years), mean (range)			56 (27-83)
	**Relationship with the primary participant, n** **(%)**			
		Spouse	17 (59)	
		Child	3 (10)	
		Sibling	2 (7)	
		Parent	2 (7)	
		Friend	5 (17)	

The sample of patients who completed the interviews was relatively balanced in terms of gender and included patients with ages ranging from their late 20s to early 80s. Importantly, there was a mix of patients who were being treated with curative intent, as well as those being treated noncuratively for de novo or recurrent metastatic disease, suggesting a wide range of cancer experiences. In terms of friends and family interviewed, ages were similar to patients, likely reflecting the high number of spouses who were included in the interviews.

### The Grounded Theory of Information Access and Use

The primary finding, or core variable [51], was that the quality of cancer-related data (CRD) that patients and their friends and family received impacted their ability to plan their lives around the consequences of the malignancy diagnosis. The theory consists of 4 interrelated concepts: (1) personal projects, (2) the cancer project, (3) information as the process of receiving and interpreting data to inform action, and (4) the quality of CRD received.

**Figure 1:**
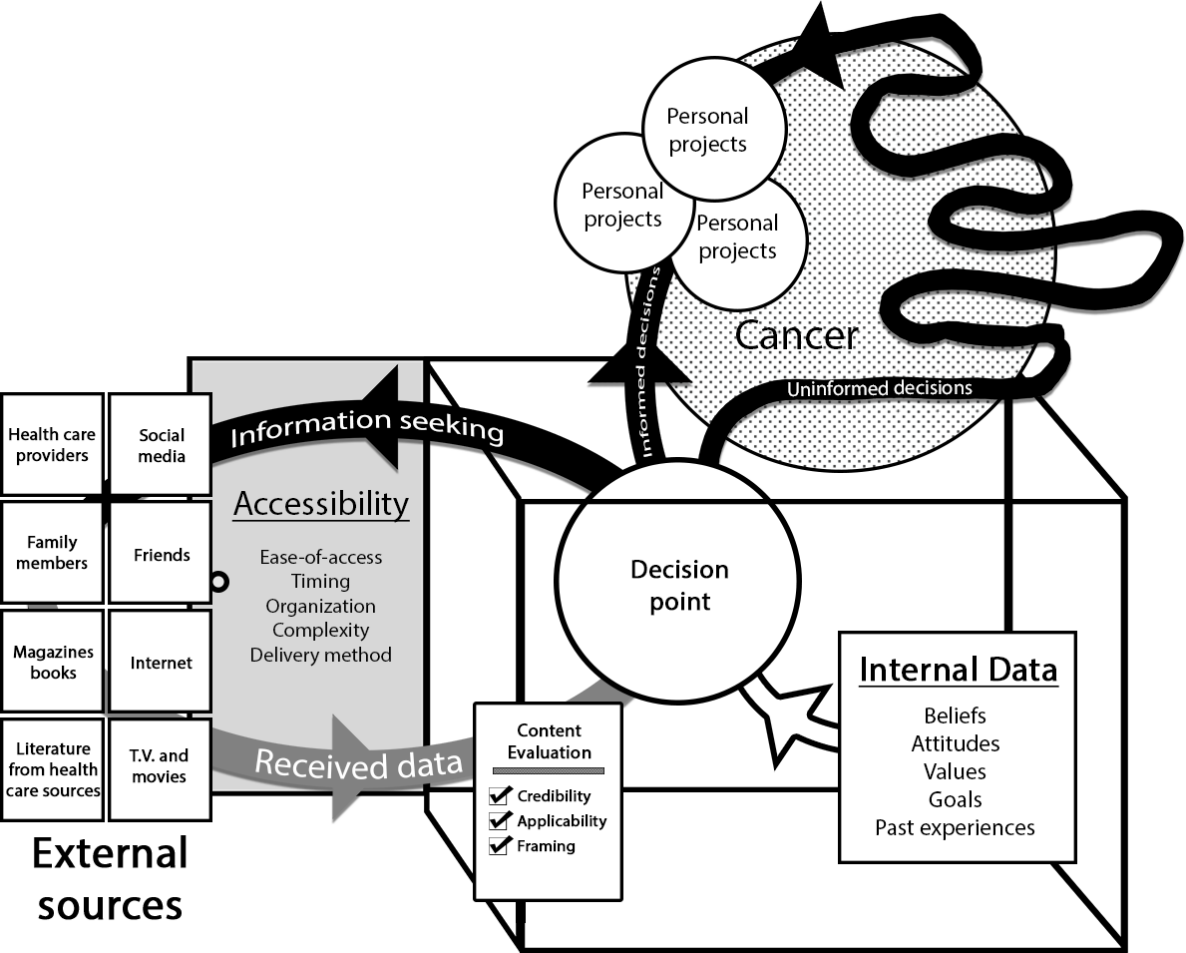
A graphical model of the theory of information access and use. The theory suggests that a cancer diagnosis is disruptive, in part, because it decreases the ability of an individual to effectively make decisions about how to invest their time and energy into their personal projects. This is due to a resulting lack of certainty about what to expect, both in terms of the cancer itself and how the consequences of cancer, such as treatment and altered life expectancy, will affect their personal projects. Information is understood as a process involving receiving data about cancer and interpreting it to increase the certainty of the outcome of different actions. High quality data, defined as data that is accessible, credible, applicable, and positively framed, enhances decision-making support and results in improved engagement with personal projects. The dark black arrowed lines represent aspects of the information/action continuum in which energy and time is diverted between personal projects and seeking information. The indirect winding line titled “uninformed decisions” illustrates the inefficient use of an individual’s finite energy and time when decisions are not informed by high-quality cancer data.

At the conclusion of this study, information was understood as the process of informing action based on the CRD that patients and their friends and family received about cancer. For those affected by cancer, CRD came from multiple sources, including health care providers, family, friends, and the internet. CRD from health care providers were the most credible and applicable; however, access to health care providers was often limited to clinical visits where the uptake of the CRD was limited. CRD found on the internet were readily accessible and provided an opportunity for repeated access. Received CRD are interpreted in the context of internal data, including the individual’s personal values, how the individual understands their life story, goals for the future, and previously obtained CRD. This process informs the individual’s actions related to managing cancer or their personal projects (eg, career, raising children, being physically fit). The concepts that comprise the theory are described in the following subsections. A graphical model of the theory is presented in Figure 1.

#### Personal Projects: The Context of Cancer

Personal projects refer to the collection of activities that an individual invests a significant amount of energy and time in over a prolonged period of their life. The following quote demonstrates how time and energy shift from one personal project (eg, child rearing) to another (eg, the project of being healthy) over the course of a lifetime:

I worked and I [raised] the kids and I was very involved, and I volunteered a ton and then once they graduated it was my turn. And I started to do things for me and get myself healthy and eat healthy and all that.

Patient 1

The personal projects of the participants created a unique context for each individual to face the challenges of the cancer experience. Each participant had a unique group of ongoing personal projects, allotting different amounts of time and energy to each, and each was ascribed with unique meaning. Importantly, before diagnosis, these personal projects—and the activities and roles that comprised them—were what the participants invested their time and energy in.

#### Cancer: A New Disruptive Project

The personal projects of the participants were disrupted following the malignancy diagnosis. Energy and time were diverted from preexisting personal projects to managing the consequences of the cancer diagnosis. Some participants described being able to continue with most aspects of their personal projects but were unable to plan how these would fit into the future. This was because the necessary details that would facilitate planning, such as prognosis or the time and energy commitment needed for treatment, were not made clear for weeks, or even months, after the initial diagnosis. Others described that following the diagnosis, their personal projects essentially halted:

I was given the diagnosis, sent to the [hospital ward] and then I was in there for over I think it was two weeks or something or longer.

Patient 15

For friends and family, disruption to personal projects was related to the amount of support they provided to the individual who received the malignancy diagnosis. A partner of a patient described the disruptive effect of cancer as follows:

Well obviously, it’s been life altering. I guess it’s – it’s certainly changed what the priorities are in our short term, midterm and long-term activities. …because like the first priority is always caring for [spouse] making sure [they] get the right [treatment].

Friends and Family 15

In this example, priorities were understood to relate to the relative energy and time that the participant planned to invest in various projects. In contrast, a participant identified as a friend to a primary participant but not as a central member of the patient’s support network indicated that the malignancy diagnosis was not disruptive to their personal projects stating:

I avoid the issue of [their] illness. …I don’t make [them] sick.

Friends and Family 26

#### Information: Definition and Function

At the conclusion of this study, information came to be understood as a multistage process, with steps occurring both externally and internally to the individual. The process participants described included searching for, receiving, and interpreting data related to cancer (ie, CRD) to inform action related to their personal projects and managing cancer. CRD came in many forms, including through conversations with health care providers, friends and family, web searches, and personal experiences. Participants also described actively searching for CRD, using time and energy that would otherwise be used for their personal projects, to understand the diagnosis and its consequences. For instance, one participant described searching for CRD and using them to answer questions related to the project of raising a family following the malignancy diagnosis:

But at the start, ‘cause I was so scared, I thought “oh my God, I have these little kids that I have to raise” you know? I have a long haul ahead of me, like I got to get through this. What can I do that’s going to help benefit me in the long run? [...] what things are going to benefit me health wise? That’s going to… help me get through this?

Patient 2

This participant went on to describe finding books and other resources that contained CRD that were helpful in navigating the challenges of being a parent while dealing with the consequences of the malignancy.

In contrast to the CRD that were actively sought out, CRD were also obtained passively through sources such as TV shows, news, and casual conversations with friends and family and from clinical encounters with physicians and nurses. In addition, participants indicated that although much of the CRD that they used were obtained from external sources, they also identified internal sources of CRD. For instance, personal knowledge gained from previous cancer experiences with family and friends was identified as an important source of CRD.

After CRD were accessed, participants described using it to guide both small-scale decisions, such as the day-to-day logistical coordination of the activities related to a single personal project (eg, taking time off work to provide transportation to a patient), and large-scale decisions, such as those that would affect all of an individual’s personal projects:

[...] if I had had a better idea about what the progression, the path [forward] is going to be - that would be helpful for me. [...] And I want to plan, right now I want to plan 6 months, I want to plan a year from now. [...] Because of this situation I’m going to be leaving my job and [moving] and to the extent possible I’d like to know, this sounds terribly selfish, but there’s a little bit of: how does this affect me?

Friends and Family 27

#### Quality CRD: Accessibility, Credibility, Applicability, and Framing

To understand how information could be optimally provided to those affected by cancer, many of the study interviews included a focus on how CRD had been provided through the cancer center or by clinicians, whether these CRD were helpful, and discussions about how CRD could be better provided. Through this exploration, and the many contrasting examples provided by the participants, 4 themes defining the quality of CRD were identified: accessibility, credibility, applicability, and framing.

##### Accessibility

Accessibility refers to how CRD are made available to individuals and is characterized by *ease of access, timing, organization, complexity,* and *delivery method*.

#### Ease of Access

Some external CRD sources can have limited accessibility, whereas others can be repeatedly and conveniently accessed. Health care providers and cancer centers are sources of CRD that have limited access. Participants described CRD available through clinic visits, support groups, and education sessions as being accessible only in certain locations and at certain times. As a result, the CRD provided were not always able to be effectively received. One participant described the experience of comparing the details that they recalled from clinician visits with a friend who had been present for the visits and had been taking notes:

I still haven’t been to a single appointment without bringing someone with me. [...] every single car ride home [when we discuss the appointment] it was like we were in two different [doctor’s visits].

Patient 30

Resources that could be accessed repeatedly, such as printed materials or the internet, allowed participants to access and interpret CRD at their own pace. The internet was a highly accessible source of CRD for many participants. One patient participant described being confused about the prognosis of their breast cancer despite receiving prognostic CRD from a physician. The participant used Google to gather additional CRD as the physician was not readily available to provide further clarification. The participant eventually decided to accept the treatment that had been recommended after learning through the internet that their breast cancer is a “more aggressive type so that’s kind of scary, but then there’s the treatment for a year that’s supposed to balance it out” (Patient 29).

#### Timing

For CRD sources with limited access, participants needed to be able to receive them when they were available. Timing refers to issues where individuals are simply not able to receive CRD even if they are physically presented with CRD. One friend and family participant described an instance of poor timing when CRD was shared with a loved one who was in the hospital and recovering from cancer surgery:

Well, he was on hard drugs. So I couldn’t – like he is stoned, when he was in the hospital. And they are throwing a ton of information at him that I am having a hard time grasping and retaining and so he doesn’t have a hope in hell of getting it.

Friends and Family 3

#### Organization

Organization affects how efficiently individuals are able to identify what content of the provided CRD is relevant to them. One participant described the experience of receiving a printed package of CRD from the cancer center, and the subsequent investment of energy and time to identify what was important:

[...] it was a lot of brochures and then for me it was about weeding out what was important and relevant, so I just focused on like overall what’s going to happen with chemo and then just hone in on like [the patient’s type of cancer]. Not that anything – like it’s not like the other things aren’t relevant but [I had to focus on] what I could like absorb [and] what I needed to know.

Friends and Family 22

#### Complexity

The complexity of the CRD being shared also affected participants’ ability to interpret it. One participant described receiving CRD with technical medical content as “good, but you can only give so much to a laymen and they’re not going to understand the rest of it like, it can only be so difficult” (Friends and Family 15). Another participant described the amount of time and energy required to navigate through complicated treatment decisions that had been offered by the medical oncologists. The participant described having multiple “family group meetings” (Friends and Family 9) in which the members of the family would sit in the patient’s living room and repeatedly play the recording of the doctor’s visit, trying to understand the CRD that were shared in the consultation to make decisions both regarding medical management and how to plan their lives around the data received.

#### Delivery Method

The method of delivery was also important in terms of participants’ ability to access CRD. Different formats of delivery, such as face-to-face discussions with clinicians, education sessions at the cancer center, and internet content, resulted in differences in terms of ease of access. Participants also expressed relative differences in their comfort in each format. Text-based CRD were universally described as helpful. However, some individuals expressed issues with retrieving internet content (“I don’t do the computer” [Patient 3]) or a preference for reading things on paper as opposed to on a computer or smartphone screen.

##### Credibility

Participants described receiving CRD from their health care providers, friends, family, the internet, TV, and other cancer survivors. The usefulness of these CRD was related to the credibility (ie, reliability) of the source.

CRD received from health care providers, including handouts and brochures, were generally considered credible. Oncology specialists, including physicians and nurses, were identified as being the most credible sources of CRD. They were described as being able to anticipate questions and provide answers without even being asked, capable of providing reassurance, and answering the patient’s questions based on “where [the patient was] coming from” (Patient 7). General practitioners or family physicians were also considered credible sources; however, several participants indicated that they received little CRD about their cancer from their general practitioner. One participant indicated that they did not trust anything from the general practitioner stating that the general practitioner had “missed the diagnosis [of malignancy] for many years” (Patient 1). Although participants described various degrees of trust in internet sources of CRD, websites such as the Canadian Cancer Society’s website were identified as highly credible.

Cancer survivors, defined here as those with a personal diagnosis or the close friend or family member of someone with a diagnosis [55], were also credible sources of CRD. Survivors provided practical, real-world knowledge about how to manage the consequences of cancer. Participants described “comparing notes” (Patient 12) about where to source complementary products such as hand creams and how to plan for certain treatments. In addition, survivors who had been diagnosed with cancer decades earlier and were still alive existed as CRD that a malignancy diagnosis was not necessarily a death sentence:

Since I have seen people stay over 10 years with [specific type of cancer], I’m hoping I will stay about 10 years [...]. I met a woman who told me “oh, this is my 10th year” [...]. So I believe that if some people can survive it then I will.

Patient 10

Participants also described CRD from sources that were not credible. They described interactions with well-meaning friends and family who provided CRD about conspiracy theories and unproven controversial treatments. These examples of CRD were described as “uncomfortable noise” (Patient 30) requiring time and energy to evaluate both its credibility and how best to manage the relationship with its source.

##### Applicability

Although participants described the CRD obtained from health care providers as highly credible, the data were not always applicable. Participants described receiving general information packages about nutrition and managing side effects but finding these of limited use or even a source of potential distress. One participant who was receiving immunotherapy described receiving a list of potential side effects of treatment from the medical team providing treatment. The participant described reading through the list and feeling anxious about the potential side effects only to become frustrated when at the bottom of the list it said that immunotherapy patients should “ignore [the list of side effects] and just call the triage number” (Patient 17).

Many participants described receiving CRD from the TV and the internet. CRD from these sources presented challenges for the participants as they were a potential source of fear. One participant described being: "worried about how bad [chemotherapy] was going to be” based on “pictur[ing] it from TV and stuff. Like people just puking all the time” only to find that “nausea was hardly a problem" [Patient 31].

Personal experience provided a source of internal CRD considered to be extremely applicable. Participants, including patients and those supporting them, described that as they gained personal experience with receiving medical care, they were able to find a “rhythm” (Patient 31) as they knew what to expect. This allowed them to become increasingly able to plan activities related to their personal projects, such as their work or other relationships. However, each new challenge, such as an unfamiliar treatment, procedure, or symptom, had the potential to interrupt this rhythm, causing disruption until a new rhythm could be established.

##### Framing

Whether CRD were framed in a *positive* way also affected participants’ ability to use the data. *Positive framing* involves communicating information in an honest manner that (1) also highlights the best possible outcome including exceptional outliers and (2) provides options for moving forward. Even when the odds were seemingly against them, participants stressed the importance of focusing on positive outcomes and what they, or clinicians, could do to optimize the situation:

Maybe they’re not right with me. Maybe I’m one of the 5%, because I exercise or whatever... I can pretend that maybe I’m one of the [few] that will beat this, to some degree, not beat it forever, but go a little longer than they told me.

Patient 1

You don’t want someone telling you, you’re for sure going to have, you know, a really bad rash on your hands and feet. You want someone saying, you might have a bad rash on your feet **and this is what you do about it.**

Patient 2

They’ve been amazing, everybody. And helpful, and encouraging. [...] They haven’t been negative about [it], they’ve just said that there is no cure for this **yet.**

Patient 8

I guess the negative part to me is – cause I’ve heard and seen people that [say] “well I have cancer so I’m going to die, I know that whether it’s five years down the road, I’m going to die.” [...] The negative part is “I’m going to die” you know?

Patient 21

## Discussion

### Interpretation of Findings

A classic definition of information is “a difference in matter-energy which affects uncertainty in a situation where a choice exists among a set of alternatives” [29,56]. Benner [57] suggests that illness and losses such as death “can disrupt (if not shatter) one’s taken-for-granted world” and that recovery comes both from “curing the body” [57] and through (re)integration of the self into “his or her particular world” [57]. The theory that emerged in this study links an individual’s ability to remain integrated in a world changed by cancer, through maintenance of connection with personal projects to their ability to access helpful information in a way that does not result in further disruption to their life.

The risk benefit consideration identified in existing theories, such as the HIA [29] and the theoretical framework of HISB by Longo et al [47], identifies that an important step in the information process is deciding whether additional information should be sought (ie, cost-benefit analyses). Similarly, this study identified that the cost of seeking CRD is two-fold. First, seeking CRD was an activity that diverted time and energy from personal projects. Second, the cost of basing expectations and making decisions on CRD that are inaccurate is not negligible, as exemplified by the quotes provided in the Applicability subsection in the *Results* section. Interventions structured on the 4 components of high-quality CRD outlined in this study, including accessibility, credibility, applicability, and framing, are likely to minimize the cost and maximize the benefit of health information seeking for those living with cancer.

#### Extending the Theory—A Deeper Grounding of Accessibility and Time

An important component of the information process, in addition to seeking, receiving, and ultimately acting on CRD, occurs between when CRD are received and action occurs. Although the process of interpreting CRD to inform action was not explored explicitly in this study, insights can be gained by examining the findings of this study in conjunction with the existing literature. First, participants in this study indicated that some of their decisions regarding treatment and their personal lives were based on limited or inaccurate CRD. Second, as participants shared and reflected on their cancer journey they both reflected feeling and displayed anger, sadness, joy, and a wide range of other emotions. Both observations are congruent with the existing literature regarding the challenges individuals living with cancer face in obtaining useful information [1-4,9,11,12,14,15,38,39,42,50] and the significant role emotions play during the cancer experience [58,59]. The role of emotions in decision making has been well documented, with both theory and empirical data supporting that emotions affect decision making in different ways [60,61]. For instance, fear is associated with the interpretation of greater risk, whereas anger is associated with less perceived risk [60,61]. In addition, research supports that individuals revert to less emotional states as time passes from the inciting stressor, resulting in decision making that is less reactive, and instead guided by reasoning that is more rational, better reflecting the individual’s personal values [60,62].

The insights gained from the data regarding emotions and decision making is important because they add depth to the concept of accessibility and the subconcept of timing that emerged from this theory. On the basis of the theoretical and empirical data regarding emotion and decision making [60,61], for CRD to be useful, they must be provided well in advance of decision making to increase the probability that decision making will be interpreted in a nonemotionally heightened state. Although there is limited empirical evidence to support this conclusion in the cancer context, the literature regarding patient decision aids (PDAs) is informative. PDAs consist of questionnaires or informational packages provided to patients to assist them in better understanding and engaging with medical decision making [19,63]. In a recent Cochrane Systematic Review, although not directly compared in any of the studies reviewed, PDAs provided before consultation compared with usual care appeared to have a positive impact on patient’s accurate perception of risk compared with PDAs provided during consultation (risk ratio 2.25, 95% CI 1.65-3.07 vs risk ratio 1.79, 95% CI 1.28-2.52, respectively) [19]. If fear is indeed associated with inaccurate risk perception [60,61], then this trend suggests that endeavors to understand the role of early provision of high-quality CRD to patients with cancer and those supporting them, guided by the intention of reducing fear and improving decisional quality, may be fruitful.

#### From Theory to Innovation

The theory that emerged in this study is useful because it identifies guiding concepts for developing high-quality CRD. Providing CRD that are accessible, credible, applicable, and positively framed is predicted to minimize the cost and maximize the benefit of information seeking. On the basis of what was shared by the participants in the interviews and the resulting theory, it is expected that the internet will be the primary delivery method of any novel informational intervention informed by this study. Although universal access to the internet is not a reality, with barriers to access existing for some groups such as those of low socioeconomic status [64], it is estimated that approximately 90% of North Americans have internet access [65], with rates of internet usage in seniors (aged 65 years and older) being over 70% in some areas [65]. As identified in this study and in the HISB literature in general [66,67], the internet circumnavigates common issues with accessibility, such as the need to travel or being available only during business hours and the requirement of appointment times to receive information [67]. In other words, it facilitates access to CRD in a way that minimizes the cost to the individual and their personal projects.

Perhaps the biggest challenge with providing highly accessible internet-based CRD is ensuring that it is adequately applicable to “assist with anticipating the sequence of events that will likely take place” [27]. It is plausible that an inversely proportional relationship exists between applicability and accessibility. For instance, in this study health care providers were identified as providing the most applicable information, yet they could only be accessed through an appointment taking place at the cancer center. The internet, on the other hand, was very accessible, but many participants identified not being sure of what information was relevant to them. Similar findings have been reported elsewhere [68,69]. Taken together with the theory that emerged, this relationship suggests that any novel online informational intervention should be integrated with and informed by local clinical practice patterns.

Given the current state of oncology practice, developing informational interventions that deliver high-quality CRD is likely possible. Contemporary clinical oncology practice relies on evidence-based, guideline-informed practice. A recent retrospective analysis of the Surveillance, Epidemiology, and End Results Program-Medicare database identified deviations from guideline recommendations in the metastatic breast cancer setting occurring only 18% of the time [70]. Similar findings in the early breast cancer population have been observed [71], supporting that, at least within the breast cancer context, care is relatively standardized in many centers. Standardization of care means that informational content can be developed that is capable of being both applicable to those in any given cancer context, providing information that helps them predict what to expect. This is because standardization likely facilitates the production of informational content that can be specific about what is going to happen regarding any given process. In contrast, when there is little standardization, specific management details, such as which clinicians will be involved, the treatments that will likely be offered, or the timing of these treatments, may quickly become inaccurate or unreliable, resulting in confusion and distress for individuals using those details to plan their lives. In addition to standardization, internet-based patient portals, which connect patients with cancer to their health care data such as consultation reports, imaging, laboratory values, and informational support, are being established at an increasing number of cancer centers [66,72-74]. This also supports that clinical integration of online informational resources delivering high-quality CRD is possible. Given that the hurdles of applicability and accessibility can be overcome, understanding how best to meet specific content needs is an important next step on the path to improving the cancer experience.

### Clinical Implications

The theory that emerged in this study informs current clinical practice in several ways. First, it highlights that clinicians are an important source of CRD. The CRD they provide is considered to be both highly credible [68] and applicable by those affected by cancer. However, the observed limitations associated with health care providers as an information source include accessibility and framing. Clinicians are encouraged to be mindful of *overloading* patients and their informal caregivers. The theory that emerged here supports that providing CRD to patients when they are emotionally overwhelmed, physically exhausted, or impaired by medication is not effective. The concept of accessibility highlights one benefit of patients having access to recordings of their visits with health care providers [75], as this intervention allows the CRD shared by health care providers to be carefully reviewed at a time that best suits the patient and their informal caregivers. With regard to framing, it has been reported elsewhere that identifying what the clinician can do for the patient, including treatment options, is an important aspect of sharing bad news [76-78]. On the basis of the findings of this study, clinicians are also encouraged, when appropriate, to empower patients and informal caregivers following bad news discussions by helping them identify ways to help themselves. This may include assisting with realistic goal setting and identifying activities that the patient and informal caregivers can engage in that will improve their situation in a meaningful way, whether directly related to the disease outcome or not.

### Research Implications

It is anticipated that this theory will be useful in guiding the development of novel interventions by providing a framework of key considerations for maximizing the benefit of information seeking in the cancer context. However, it does not provide explicit guidance on content to be included in a novel resource or the format of that content. Although the information needs of those affected by cancer are well characterized, it is not clear from the literature what specific content and resources would be most helpful for meeting those needs. Researchers are encouraged to build on this study and engage with those affected by cancer as partners [79] to systematically identify the content and method of delivery that is most helpful to those navigating the cancer journey, both in general and in specific contexts not limited to geography, culture, age, gender, sexuality, education, and income.

### Limitations

This theory was generated by engaging with adult patients and their friends and family, without exclusion on the basis of cancer type, stage, or treatment intent. The sampling approach focused on obtaining diverse, contrasting perspectives and experiences [51,80]. It is expected that, through the constant comparative method used in this grounded theory study, the emerging theoretical framework will be applicable across the general cancer context. However, specific demographic and cultural groups were not focused on, as this was not within the scope of this study. It is certainly plausible that the concepts, such as the components of high-quality CRD that emerged from this study will be of varying relevance in different populations. For instance, it is plausible that accessibility may be more important for persons with hectic schedules—such as young adults balancing establishing a career, growing a young family, maintaining a social schedule, and facing the challenges of a new cancer diagnosis—compared with individuals with fewer competing commitments. In addition, although the interpretation of CRD is presented here as a personal process, it has been demonstrated that some individuals may prefer to involve various members of their community, including elders, extended family, and/or spiritual leaders in decision making [81,82]. These 2 observations serve as a reminder that theory is not a substitute for engaging with the expected end users when developing interventions intended to help improve their cancer experience [83]. Finally, as this research study was conducted in the context of the cancer experience in Western Canada, it is certainly possible that how this framework applies to other areas in Canada, and the world for that matter, may differ. Researchers and clinicians are encouraged to explore how the framework presented here can be modified to best reflect the context of living with cancer in their area [23].

### Conclusions

The objective of this study is to develop a theoretical framework grounded in the cancer experience capable of guiding the development of informational resources. The framework that emerged links the quality of CRD received to the impact that the cancer diagnosis has on an individual’s life. The theory comprises 4 variables: personal projects, cancer as a project that interferes with existing personal projects, information as the process of receiving and processing CRD to inform action, and CRD quality. Key features of high-quality CRD include accessibility, credibility, applicability, and framing. On the basis of this theory, the internet is foundational for delivering highly accessible information interventions. Clinicians are encouraged to consider accessibility and framing in how they provide information to those they care for. Future directions for research are expected to include engaging with those affected by cancer as partners to develop and integrate informational interventions based on this theory into clinical care. Interventions informed by this theoretical work are expected to help individuals remain effectively engaged with the personal projects in their lives following a cancer diagnosis and minimize the disruptive impact of the cancer diagnosis on patients and their informal caregivers by decreasing the cost of obtaining useful information.

## Appendix

Multimedia Appendix 1 Demographic or intake questionnaire used to guide selection of interview participants.

Multimedia Appendix 2 Initial interview guide for semistructured interviews.

## Abbreviations

CGT: classic grounded theory
CRD: cancer-related data
HIA: health information acquisition
HISB: health information–seeking behavior
MBSS: Miller Behavioral Style Scale
PDA: patient decision aid

## References

[1] Tariman JD, Doorenbos A, Schepp KG, Singhal S, Berry DL (2014). Information needs priorities in patients diagnosed with cancer: a systematic review. J Adv Pract Oncol.

[2] Fletcher C, Flight I, Chapman J, Fennell K, Wilson C (2017). The information needs of adult cancer survivors across the cancer continuum: a scoping review. Patient Educ Couns.

[3] Mistry A, Wilson S, Priestman T, Damery S, Haque M (2010). How do the information needs of cancer patients differ at different stages of the cancer journey? A cross-sectional survey. JRSM Short Rep.

[4] Rutten LJ, Arora NK, Bakos AD, Aziz N, Rowland J (2005). Information needs and sources of information among cancer patients: a systematic review of research (1980-2003). Patient Educ Couns.

[5] Wang T, Molassiotis A, Chung BP, Tan J (2018). Unmet care needs of advanced cancer patients and their informal caregivers: a systematic review. BMC Palliat Care.

[6] Lambert SD, Harrison JD, Smith E, Bonevski B, Carey M, Lawsin C, Paul C, Girgis A (2012). The unmet needs of partners and caregivers of adults diagnosed with cancer: a systematic review. BMJ Support Palliat Care.

[7] Moghaddam N, Coxon H, Nabarro S, Hardy B, Cox K (2016). Unmet care needs in people living with advanced cancer: a systematic review. Support Care Cancer.

[8] Bonacchi A, Di Miceli S, Lippi D, Muraca MG, Miccinesi G (2018). Unmet needs of Italian cancer patients in different stages of the disease and care process. Tumori.

[9] Halkett GK, Kristjanson LJ, Lobb E, O'Driscoll C, Taylor M, Spry N (2010). Meeting breast cancer patients' information needs during radiotherapy: what can we do to improve the information and support that is currently provided?. Eur J Cancer Care (Engl).

[10] Hubbeling HG, Rosenberg SM, González-Robledo MC, Cohn JG, Villarreal-Garza C, Partridge AH, Knaul FM (2018). Psychosocial needs of young breast cancer survivors in Mexico City, Mexico. PLoS One.

[11] McCarthy MC, McNeil R, Drew S, Orme L, Sawyer SM (2018). Information needs of adolescent and young adult cancer patients and their parent-carers. Support Care Cancer.

[12] Meredith C, Symonds P, Webster L, Lamont D, Pyper E, Gillis CR, Fallowfield L (1996). Information needs of cancer patients in west Scotland: cross sectional survey of patients' views. Br Med J.

[13] Preisler M, Rohrmoser A, Goerling U, Kendel F, Bär K, Riemer M, Heuse S, Letsch A (2019). Early palliative care for those who care: a qualitative exploration of cancer caregivers' information needs during hospital stays. Eur J Cancer Care (Engl).

[14] Vivar CG, McQueen A (2005). Informational and emotional needs of long-term survivors of breast cancer. J Adv Nurs.

[15] Boons CC, Harbers L, Timmers L, de Jong J, Swart EL, Harry Hendrikse N, Janssen JJ, Hugtenburg JG (2018). Needs for information and reasons for (non)adherence in chronic myeloid leukaemia: be aware of social activities disturbing daily routines. Eur J Haematol.

[16] Arthurs G, Simpson J, Brown A, Kyaw O, Shyrier S, Concert CM (2015). The effectiveness of therapeutic patient education on adherence to oral anti-cancer medicines in adult cancer patients in ambulatory care settings: a systematic review. JBI Database System Rev Implement Rep.

[17] Veroff D, Marr A, Wennberg DE (2013). Enhanced support for shared decision making reduced costs of care for patients with preference-sensitive conditions. Health Aff (Millwood).

[18] Walsh T, Barr PJ, Thompson R, Ozanne E, O'Neill C, Elwyn G (2014). Undetermined impact of patient decision support interventions on healthcare costs and savings: systematic review. Br Med J.

[19] Stacey D, Légaré F, Lewis K, Barry MJ, Bennett CL, Eden KB, Holmes-Rovner M, Llewellyn-Thomas H, Lyddiatt A, Thomson R, Trevena L (2017). Decision aids for people facing health treatment or screening decisions. Cochrane Database Syst Rev.

[20] Ferrari M, Ripamonti CI, Hulbert-Williams NJ, Miccinesi G (2019). Relationships among unmet needs, depression, and anxiety in non-advanced cancer patients. Tumori.

[21] Ugalde A, Aranda S, Krishnasamy M, Ball D, Schofield P (2012). Unmet needs and distress in people with inoperable lung cancer at the commencement of treatment. Support Care Cancer.

[22] Thiessen M, Hack TF, Pitz M, Anderson M (2018). A model of identity grounded in the acute season of survivorship. Psychooncology.

[23] Glaser BG, Strauss AL (1967). The Discovery of Grounded Theory.

[24] Holton J, Walsh I (2016). Classic Grounded Theory: Applications With Qualitative and Quantitative Data.

[25] Savolainen R (2019). Modeling the interplay of information seeking and information sharing. Aslib J Inf Manag.

[26] Zare-Farashbandi F, Lalazaryan A (2014). A Review of models and theories of health information seeking behavior. Int J Health Syst Disaster Manage.

[27] Lambert SD, Loiselle CG (2007). Health information seeking behavior. Qual Health Res.

[28] Miller SM (1987). Monitoring and blunting: validation of a questionnaire to assess styles of information seeking under threat. J Pers Soc Psychol.

[29] Freimuth V, Stein J, Kean T (1989). Searching for Health Information: The Cancer Information Service Model.

[30] Rees C, Bath P (2000). The psychometric properties of the miller behavioural style scale with adult daughters of women with early breast cancer: a literature review and empirical study. J Adv Nurs.

[31] Kola S, Walsh JC, Hughes BM, Howard S (2013). Matching intra-procedural information with coping style reduces psychophysiological arousal in women undergoing colposcopy. J Behav Med.

[32] Miller SM (2015). Monitoring processing style: to see or not to see. Eur J Pain.

[33] Biernatzki L, Kuske S, Genz J, Ritschel M, Stephan A, Bächle C, Droste S, Grobosch S, Ernstmann N, Chernyak N, Icks A (2018). Information needs in people with diabetes mellitus: a systematic review. Syst Rev.

[34] Carey M, Lambert S, Smits R, Paul C, Sanson-Fisher R, Clinton-McHarg T (2012). The unfulfilled promise: a systematic review of interventions to reduce the unmet supportive care needs of cancer patients. Support Care Cancer.

[35] Clarke MA, Moore JL, Steege LM, Koopman RJ, Belden JL, Canfield SM, Meadows SE, Elliott SG, Kim MS (2016). Health information needs, sources, and barriers of primary care patients to achieve patient-centered care: a literature review. Health Informatics J.

[36] Faury S, Koleck M, Foucaud J, M'Bailara K, Quintard B (2017). Patient education interventions for colorectal cancer patients with stoma: a systematic review. Patient Educ Couns.

[37] Howell D, Harth T, Brown J, Bennett C, Boyko S (2017). Self-management education interventions for patients with cancer: a systematic review. Support Care Cancer.

[38] Hyun YG, Alhashemi A, Fazelzad R, Goldberg AS, Goldstein DP, Sawka AM (2016). A systematic review of unmet information and psychosocial support needs of adults diagnosed with thyroid cancer. Thyroid.

[39] Kotronoulas G, Papadopoulou C, Burns-Cunningham K, Simpson M, Maguire R (2017). A systematic review of the supportive care needs of people living with and beyond cancer of the colon and/or rectum. Eur J Oncol Nurs.

[40] Qan'ir Y, Song L (2019). Systematic review of technology-based interventions to improve anxiety, depression, and health-related quality of life among patients with prostate cancer. Psychooncology.

[41] Jacobs W, Amuta AO, Jeon KC (2017). Health information seeking in the digital age: an analysis of health information seeking behavior among US adults. Cogent Social Sciences.

[42] Saab MM, Reidy M, Hegarty J, O'Mahony M, Murphy M, Von Wagner C, Drummond FJ (2018). Men's information-seeking behavior regarding cancer risk and screening: a meta-narrative systematic review. Psychooncology.

[43] Rutherford C, Mercieca-Bebber R, Butow P, Wu JL, King MT (2017). Treatment decision-making in ductal carcinoma in situ: a mixed methods systematic review of women's experiences and information needs. Patient Educ Couns.

[44] Heath G, Cooke R, Cameron E (2015). A theory-based approach for developing interventions to change patient behaviours: a medication adherence example from paediatric secondary care. Healthcare (Basel).

[45] Craig P, Dieppe P, Macintyre S, Michie S, Nazareth I, Petticrew M, Medical Research Council Guidance (2008). Developing and evaluating complex interventions: the new medical research council guidance. Br Med J.

[46] Michie S, Prestwich A (2010). Are interventions theory-based? Development of a theory coding scheme. Health Psychol.

[47] Longo DR, Ge B, Radina ME, Greiner A, Williams CD, Longo GS, Mouzon DM, Natale-Pereira A, Salas-Lopez D (2013). Understanding breast-cancer patients' perceptions: health information-seeking behaviour and passive information receipt. J Commun Healthcare.

[48] Longo DR (2005). Understanding health information, communication, and information seeking of patients and consumers: a comprehensive and integrated model. Health Expect.

[49] Longo DR, Patrick TB, Kruse RL (2001). The natural history of the use of healthcare information by women with breast cancer: a conceptual model. Proc AMIA Symp.

[50] Galloway S, Graydon J, Harrison D, Evans-Boyden B, Palmer-Wickham S, Burlein-Hall S, Rich-van der Bij L, West P, Blair A (1997). Informational needs of women with a recent diagnosis of breast cancer: development and initial testing of a tool. J Adv Nurs.

[51] Glaser B, Strauss A (2009). The Discovery of Grounded Theory.

[52] Suzana Mediani H (2017). An introduction to classical grounded theory. SOJ Nurs Health Care.

[53] Rieger KL (2019). Discriminating among grounded theory approaches. Nurs Inq.

[54] Simmons O (2011). Why Classic Ground Theory.

[55] Twombly R (2004). What's in a name: who is a cancer survivor?. J Natl Cancer Inst.

[56] Rogers E, Kincaid D (1981). Communication Networks:Communication Networks: Toward a New Paradigm for ResearchToward a New Paradigm for Research.

[57] Benner P, Toombs S (2001). The Phenomenon of Care. Handbook of Phenomonology and Medicine.

[58] Jagannathan A, Juvva S (2016). Emotions and coping of patients with head and neck cancers after diagnosis: a qualitative content analysis. J Postgrad Med.

[59] Thornton LM, Levin AO, Dorfman CS, Godiwala N, Heitzmann C, Andersen BL (2014). Emotions and social relationships for breast and gynecologic patients: a qualitative study of coping with recurrence. Psychooncology.

[60] Lerner JS, Li Y, Valdesolo P, Kassam KS (2015). Emotion and decision making. Annu Rev Psychol.

[61] Mazzocco K, Masiero M, Carriero MC, Pravettoni G (2019). The role of emotions in cancer patients' decision-making. Ecancermedicalscience.

[62] McAlpine K, Lewis KB, Trevena LJ, Stacey D (2018). What is the effectiveness of patient decision aids for cancer-related decisions? A systematic review subanalysis. JCO Clin Cancer Inform.

[63] DrugTherapeutics Bulletin (2013). An introduction to patient decision aids. Br Med J.

[64] McCloud RF, Okechukwu CA, Sorensen G, Viswanath K (2016). Beyond access: barriers to internet health information seeking among the urban poor. J Am Med Inform Assoc.

[65] Statistics C (2020). Canadian Internet Use Survey. Onlin.

[66] Ashkanani H, Asery R, Bokubar F, AlAli N, Mubarak S, Buabbas A, Almajran A (2019). Web-based health information seeking among students at Kuwait university: cross-sectional survey study. JMIR Form Res.

[67] van der Maas M, Shi J, Elton-Marshall T, Hodgins DC, Sanchez S, Lobo DS, Hagopian S, Turner NE (2019). Internet-based interventions for problem gambling: scoping review. JMIR Ment Health.

[68] Sbaffi L, Rowley J (2017). Trust and credibility in web-based health information: a review and agenda for future research. J Med Internet Res.

[69] Rolfe A, Cash-Gibson L, Car J, Sheikh A, McKinstry B (2014). Interventions for improving patients' trust in doctors and groups of doctors. Cochrane Database Syst Rev.

[70] Williams CP, Azuero A, Kenzik KM, Pisu M, Nipp RD, Bhatia S, Rocque GB (2019). Guideline discordance and patient cost responsibility in medicare beneficiaries with metastatic breast cancer. J Natl Compr Canc Netw.

[71] Williams CP, Kenzik KM, Azuero A, Williams GR, Pisu M, Halilova KI, Ingram SA, Yagnik SK, Forero A, Bhatia S, Rocque GB (2019). Guideline discordance and patient cost responsibility in medicare beneficiaries with metastatic breast cancer. Oncologist.

[72] Han H, Gleason KT, Sun C, Miller HN, Kang SJ, Chow S, Anderson R, Nagy P, Bauer T (2019). Using patient portals to improve patient outcomes: systematic review. JMIR Hum Factors.

[73] Pho K, Lu R, Gates S, Xie Y, Lee SJ, Gerber DE (2018). Characteristics of patients using patient portals in oncology. JAMA Oncol.

[74] Alpert JM, Morris BB, Thomson MD, Matin K, Brown RF (2018). Implications of patient portal transparency in oncology: qualitative interview study on the experiences of patients, oncologists, and medical informaticists. JMIR Cancer.

[75] Hack TF, Ruether JD, Weir LM, Grenier D, Degner LF (2011). Study protocol: addressing evidence and context to facilitate transfer and uptake of consultation recording use in oncology: a knowledge translation implementation study. Implement Sci.

[76] Baile WF, Buckman R, Lenzi R, Glober G, Beale EA, Kudelka AP (2000). SPIKES-a six-step protocol for delivering bad news: application to the patient with cancer. Oncologist.

[77] Girgis A, Sanson-Fisher RW (1995). Breaking bad news: consensus guidelines for medical practitioners. J Clin Oncol.

[78] Rat A, Ricci L, Guillemin F, Ricatte C, Pongy M, Vieux R, Spitz E, Muller L (2018). Development of a web-based formative self-assessment tool for physicians to practice breaking bad news (BRADNET). JMIR Med Educ.

[79] Mallidou AA, Frisch N, Doyle-Waters MM, MacLeod ML, Ward J, Atherton P (2018). Patient-oriented research competencies in health (PORCH) for patients, healthcare providers, decision-makers and researchers: protocol of a scoping review. Syst Rev.

[80] Birks M, Mills J (2010). Grounded Theory: A Practical Guide.

[81] Dolan H, Alden DL, Friend JM, Lee PY, Lee YK, Ng CJ, Abdullah KL, Trevena L (2019). Culture, self, and medical decision making in Australia and China: a structural model analysis. MDM Policy Pract.

[82] Hawley ST, Morris AM (2017). Cultural challenges to engaging patients in shared decision making. Patient Educ Couns.

[83] Carman KL, Dardess P, Maurer M, Sofaer S, Adams K, Bechtel C, Sweeney J (2013). Patient and family engagement: a framework for understanding the elements and developing interventions and policies. Health Aff (Millwood).

