# Supplementary material for: Information Access and Use by Patients With Cancer and Their Friends and Family: Development of a Grounded Theory
Source: J Med Internet Res. 2020 Oct 29;22(10):e20510. doi: 10.2196/20510 (PMC7661235; doi:10.2196/20510)
Supplement: Multimedia Appendix 1 [file jmir_v22i10e20510_app1.docx]

Survey for Patients

We would prefer you to complete this survey at home; Please complete the survey and either email it with your signed consent form it to [maclean.thiessen@ahs.ca](mailto:maclean.thiessen@ahs.ca) or call 403-521-3276 to make other arrangement.

Date of Birth: Gender:

Postal Code:

Marital Status:

| Single | Married/Common Law | Divorced | Widowed |
| --- | --- | --- | --- |

Education Level:

| Some High School | Graduated High School | Some University/College | Graduated University/College |
| --- | --- | --- | --- |

Is English your first language?

| YES | NO |
| --- | --- |

Where you born in Canada?

| YES | NO |
| --- | --- |

If you weren’t born in Canada, how many years have you lived in Canada:

|  |
| --- |

Annual Household Income:

| Less than $20,000 | $20,000 to $50,000 | $50,000 to $100,000 | More than $100,000 |
| --- | --- | --- | --- |

I am completing this survey as a:

| A cancer patient | Friend of a cancer patient | Family of a cancer patient |
| --- | --- | --- |

Since the cancer diagnosis, has your family:

Had to alter it’s plans/goals for the future?

| YES | NO |
| --- | --- |

Been able to make new plans and goals for the future?

| YES | NO |
| --- | --- |

Had to change how duties and tasks are divided?

| YES | NO |
| --- | --- |

Been as reliable with each other as before?

(i.e. make and keep commitments to each other)

| YES | NO |
| --- | --- |

Where you given enough information **at the time of diagnosis** to plan your life around your cancer treatment?

| YES | NO |
| --- | --- |

Are there things you wish you would have been told about your diagnosis that you weren’t?

| YES | NO |
| --- | --- |

Are there things you wish you would have been told about your treatment that you weren’t?

| YES | NO |
| --- | --- |

Based on the information you have been given, what is the chance that someone with a cancer like yours will be cured?

| Not Curable | Under 25% | 25% - 75% | Above 75% | It will be cured | Not Sure |
| --- | --- | --- | --- | --- | --- |

Based on what you know, what is the average amount of time someone with a cancer like yours could expect to survive?

| Months | Years | Decades | Not Sure |
| --- | --- | --- | --- |

Survey for Family Members of Patients

We would prefer you to complete this survey at home; Please complete the survey and either email with your signed consent form it to [maclean.thiessen@ahs.ca](mailto:maclean.thiessen@ahs.ca) or call 403-521-3276 to make other arrangement.

Date of Birth: Gender: Postal Code:

Marital Status:

| Single | Married/Common Law | Divorced | Widowed |
| --- | --- | --- | --- |

Education Level:

| Some High School | Graduated High School | Some University/College | Graduated University/College |
| --- | --- | --- | --- |

Is English your first language?

| YES | NO |
| --- | --- |

Where you born in Canada?

| YES | NO |
| --- | --- |

If you weren’t born in Canada, how many years have you lived in Canada:

|  |
| --- |

Annual Household Income:

| Less than $20,000 | $20,000 to $50,000 | $50,000 to $100,000 | More than $100,000 |
| --- | --- | --- | --- |

What is your relationship to the patient: (i.e. brother, sister, son, daughter, uncle, aunt, grandmother, grandfather)

|  |
| --- |

Since the cancer diagnosis, has your family:

Had to alter it’s plans/goals for the future?

| YES | NO |
| --- | --- |

Been able to make new plans and goals for the future?

| YES | NO |
| --- | --- |

Had to change how duties and tasks are divided?

| YES | NO |
| --- | --- |

Been as reliable with each other as before? (i.e. make and keep commitments to each other)

| YES | NO |
| --- | --- |

Survey For Friends

We would prefer you to complete this survey at home; Please complete the survey and either email with your signed consent form it to [maclean.thiessen@ahs.ca](mailto:maclean.thiessen@ahs.ca) or call 403-521-3276 to make other arrangement.

Date of Birth: Gender: Postal Code:

Marital Status:

| Single | Married/Common Law | Divorced | Widowed |
| --- | --- | --- | --- |

Education Level:

| Some High School | Graduated High School | Some University/College | Graduated University/College |
| --- | --- | --- | --- |

Is English your first language?

| YES | NO |
| --- | --- |

Where you born in Canada?

| YES | NO |
| --- | --- |

If you weren’t born in Canada, how many years have you lived in Canada:

|  |
| --- |

Annual Household Income:

| Less than $20,000 | $20,000 to $50,000 | $50,000 to $100,000 | More than $100,000 |
| --- | --- | --- | --- |

I am completing this survey as a:

| A cancer patient | Friend of a cancer patient | Family of a cancer patient |
| --- | --- | --- |

How long have you known the patient:

| Less than 1 year | 1 – 5 years | 5 – 10 years | More than 10 years |
| --- | --- | --- | --- |

Since the diagnosis, has your friend (understandably) had to alter previously made commitments with you?

| YES | NO |
| --- | --- |

Since the diagnosis, has the patient been able to be as reliable regarding making and following through on plans with you?

| YES | NO |
| --- | --- |

Have you been able to be as supportive as you would have liked towards the patient, regarding their diagnosis?

| YES | NO |
| --- | --- |

Have you been able to find support to help you cope with the patient’s diagnosis?

| YES | NO |
| --- | --- |

Is there a certain type of information that you did not have access to that you think would have helped you be a more supportive friend?

| YES | NO |
| --- | --- |
