# Supplementary material for: Information Access and Use by Patients With Cancer and Their Friends and Family: Development of a Grounded Theory
Source: J Med Internet Res. 2020 Oct 29;22(10):e20510. doi: 10.2196/20510 (PMC7661235; doi:10.2196/20510)
Supplement: Multimedia Appendix 2 [file jmir_v22i10e20510_app2.docx]

Initial Sample Semi-Structured Interview Guide:

**Bolded** questions sections will be asked as appropriate. *Italic* text indicates interview script to be stated at the start and end of the interview.

*Thank you for agreeing to meet with me/visit over the phone. This interview should take approximately fourty-five minutes. It is being recorded. At any point, you are welcome to take a break or stop the interview all together.*

**Questions for patients only:**

Tell me how you learned about the cancer diagnosis?

How has your life changed since the diagnosis?

Where you prepared for these changes?

Some people have expressed that when they are diagnosed with cancer they lose the ability to plan their lives. Did you find this to be true?

Did you find that you were adequately prepared for the impact chemotherapy (and radiation is applicable) had on your life?

What do you consider your work?

How has diagnosis and treatment affected your work?

How far ahead are you making plans right now?

What kind of information helped you plan your life?

Is there any information that you did not receive that would have helped you maintain commitments to friends and family?

What about information to help you maintain your work?

When would the best time to have received this information have been?

What kind of format would have worked the best?

Would receiving detailed information about possible treatments, including schedules, benefits and side-effects and their duration have been helpful prior to meeting with your oncologist?

Would information about chance of cure and average life-expectancy been helpful to receive prior to meeting with the oncologist?

**Questions focusing on information for family (for family and patients):**

After the diagnosis, did your families routine change?

Did attending medical appointments and receiving treatment affect your families function?

How was it affected? *or* How did you manage to not let treatment and medical appointments affect your families functioning?

What kind of information did you receive that helped you maintain your family function after diagnosis and through treatment?

How have you been a supportive family member?

How have your family members been supporting you?

Is there any information that you can think of that would help you be more effective at supporting your family members?

Is there any information that would help support you?

What kind of information would have been helpful to support your family in maintaining it’s ability to function after diagnosis and through treatment?

When would the best time to receive this information be?

What would have been the best way to receive this information?

What are your thoughts on receiving this information perhaps in the mail or outside of the clinic?

**Questions for friends only:**

After the diagnosis, did you notice any changes in your relationship?

Did any of the plans you had made previously with ‘patient’ change after the diagnosis?

How have you found making plans with ‘patient’ since the diagnosis?

Have you found ways to be supportive? What kind of things to you do?

Is there any information that would help you be a more supportive friend?

Is there any information that would help support you, as the friend of someone living with cancer?

When would you have liked to receive this information?

What would be the best format for this information?

*Thanks for taking part in this interview. This concludes your participation in this study. Contact information for myself, my supervisor, and the Alberta Health Sciences Research Ethics Board are listed inside of the consent form if you have any questions, comments or concerns.*
